# Near-infrared light-triggered NO release for spinal cord injury repair

**DOI:** 10.1126/sciadv.abc3513

**Published:** 2020-09-25

**Authors:** Yaqin Jiang, Pengfei Fu, Yanyan Liu, Chaochao Wang, Peiran Zhao, Xu Chu, Xingwu Jiang, Wei Yang, Yelin Wu, Ya Wang, Guohua Xu, Jin Hu, Wenbo Bu

**Affiliations:** 1 Shanghai Key Laboratory of Green Chemistry and Chemical Processes, School of Chemistry and Molecular Engineering, East China Normal University, Shanghai 200062, P. R. China.; 2 Department of Materials Science, Fudan University, Shanghai 200433, P. R. China.; 3 Department of Neurosurgery, Huashan Hospital, Fudan University, Shanghai 200040, P. R. China.; 4 Tongji University Cancer Center, Shanghai Tenth People’s Hospital, Tongji University School of Medicine, Shanghai 200072, P. R. China.; 5 Department of Spine Surgery, Changzheng Hospital, Second Military Medical University, Shanghai 200040, P. R. China.; 6 State Key Laboratory of High Performance Ceramics and Superfine Microstructure, Shanghai Institute of Ceramics, Chinese Academy of Sciences, Shanghai 200050, P. R. China.

## Abstract

Traumatic spinal cord injury (SCI) is caused by external physical impacts and can induce complex cascade events, sometimes converging to paralysis. Existing clinical drugs to traumatic SCI have limited therapeutic efficacy because of either the poor blood–spinal cord barrier (BSCB) permeability or a single function. Here, we suggest a “pleiotropic messenger” strategy based on near-infrared (NIR)–triggered on-demand NO release at the lesion area for traumatic SCI recovery via the concurrent neuroregeneration and neuroprotection processing. This NO delivery system was constructed as upconversion nanoparticle (UCNP) core coated by zeolitic imidazolate framework–8 (ZIF-8) with NO donor (CysNO). This combined strategy substantial promotes the repair of SCI in vertebrates, ascribable to the pleiotropic effects of NO including the suppression of gliosis and inflammation, the promotion of neuroregeneration, and the protection of neurons from apoptosis, which opens intriguing perspectives not only in nerve repair but also in neurological research and tissue engineering.

## Teaser

Presenting a “pleiotropic messenger” strategy using near-infrared–triggered on-demand NO release for spinal cord injury repair.

## INTRODUCTION

Spinal cord injury (SCI) is defined as damage to spinal cord with devastating consequences for the physical, social, and vocational well-being of patients (*1*). One of the pathogenic factors is trauma, including external physical impacts such as a traffic accident, fall, sports injury, and assault (*2*, *3*). In traumatic SCI, the long axons of spinal cord neurons are usually severed, which can induce a series of complex cellular and molecular cascade events, including inflammation, neuronal damage, and death, and sometimes converging to paralysis (*4*). Activating cell differentiation and neurite growth for neuroregeneration, as well as preventing inflammation occurrence for neuroprotection are key points for efficient repair of traumatic SCI (*5*). However, these current clinical drugs can only work in one manner, either neuroprotection or neuroregeneration, of this complex and multifaceted disease (*6*). Moreover, the common existing blood–spinal cord barrier (BSCB) largely limits their efficient diffusion into spinal cord, thus leading to unsatisfied curative effects (*7*, *8*). Therefore, a multifunctional and integrated drug system is urgently needed.

Nitric oxide (NO) is an omnipresent intercellular messenger in all vertebrates, which modulates numerous physiological signaling events, such as blood flow, thrombosis, and neural activity (*9*, *10*). Although NO is often described as highly toxic and reactive, it is not when at an appropriate level (*11*). Studies have proved that NO can serve as a promoter for neural growth and regeneration (*12*), as well as a neuron protector against inflammation (*13*) and apoptosis (*14*). Notably, because of its small size and hydrophobicity, NO can diffuse rapidly across any tissues and physical barriers (e.g., blood-brain barrier and BSCB) for executive functions. Overall, NO is a potential therapeutic agent to traumatic SCI, only if this drug system is designed to achieve on-demand NO release at a certain period of time. S-nitrosothiols (RSNOs) are one class of photosensitive NO donors believed to store/transport NO in vivo for immediate NO release upon ultraviolet (UV) light stimulation, which has been used for the treatment of cancer (*15*) and inflammation (*16*). Constructing one functional system for the low–molecular weight RSNOs’ loading and the short-wavelength UV light source importing in the focus area may enable efficient NO storage and on-demand release for traumatic SCI therapy with high spatiotemporal resolution.

**Fig. 1:**
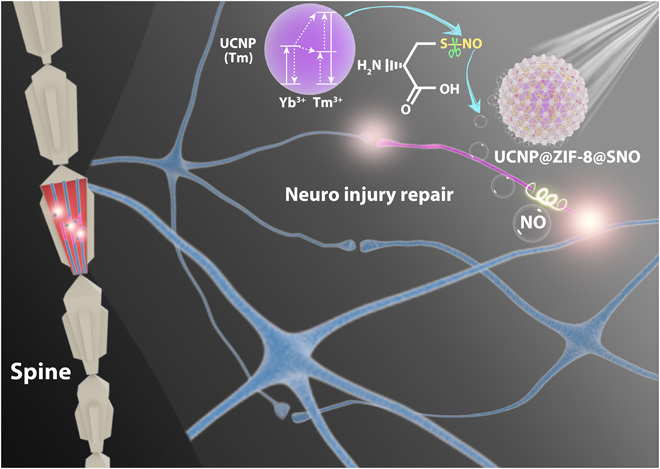
Schematic diagram of NIR-controlled NO release for SCI repair. The NO delivery nanosystem was constructed as an UCNP core coated by zeolitic imidazolate framework material (ZIF-8) shell with NO photochemical donor (CysNO). UCNPs convert NIR light to blue-violet light, which can cleave the S─NO bond in CysNO for NO release. The pleiotropic effects of NO, including the suppression of gliosis and inflammation, the promotion of neuronal regeneration, and the protection of neurons from apoptosis, facilitate the growth of injured motor neuron axons in zebrafish, as well as the recovery of the motor functions in traumatic SCI rats.

Here, we suggest a “pleiotropic messenger” strategy based on near-infrared (NIR)–triggered on-demand NO release at the lesion area for traumatic SCI recovery. This functional system was designed with upconversion nanoparticle (UCNP) coated by zeolitic imidazolate framework–8 (ZIF-8) with flexible framework for nitrosothiol (CysNO) loading (designated UCZN) (*17*, *18*). As shown in Fig. 1, upon NIR irradiation, the upconverted UV light of UCNP transfers to CysNO to cleave the S-NO bond for NO release. In vitro studies show that the released NO at an appropriate level can activate the differentiation pathway for a markedly pronounced outgrowth of neuronal processes in both PC12 cells and dorsal root ganglion (DRG) neurons. Moreover, NIR light as a noninvasive energy field exhibits biosafety and deep tissue penetration, and so can be an ideal excitation source for biological application (*19*, *20*). For in vivo experiments, UCZN after one 980-nm laser irradiation promotes the growth of the damaged motor neuron axons in zebrafish, about 42% longer than that without any treatment. This combined treatment substantially facilitates the recovery of the motor functions in Sprague-Dawley rats with traumatic SCI, with 90% reduction of the lesion area, ascribable to the pleiotropic effects of NO, including the suppression of gliosis and inflammation, the promotion of neuronal regeneration, and the protection of neurons from apoptosis. Notably, the nanosystem can stay in the lesion area and work for a long time, suggesting a possible use for repeated doses of NO in the treatment of some stubborn chronic neuropathies, such as Alzheimer’s and epilepsy.

## RESULTS

### Synthesis and characterization of UCZN

**Fig. 2:**
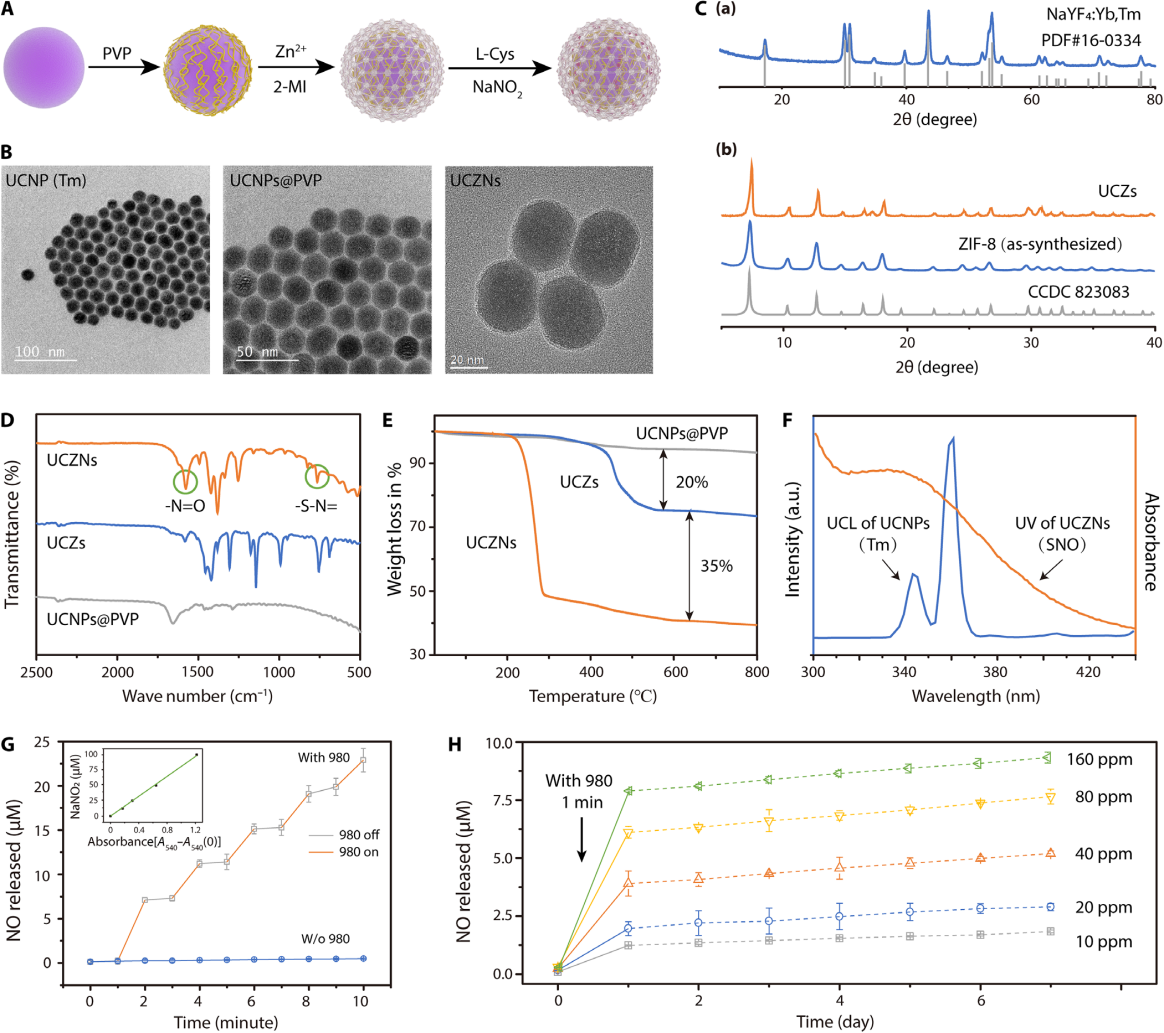
Synthesis and characterization of UCZN. (**A**) Preparation of UCZN. (**B**) TEM images of UCNPs, UCNPs@PVP, and UCNPs@PVP@ZIF-8@CysNO (UCZN). (**C**) XRD patterns of UCNPs (a) and UCNPs@PVP@ZIF-8 (UCZs) (b). (**D**) FTIR spectra of UCNPs@PVP, UCZs, and UCZN, with the characteristic peaks of RSNOs marked by the green circles. (**E**) Thermogravimetric curves of the product in each step, with the calculated difference values between them. (**F**) Fluorescence emission spectra of UCNPs and UV-Vis absorption of UCZN (wavelength from 300 to 440 nm). (**G**) Released amount of NO from UCZN [160 parts per million (ppm)] with or without NIR light activation. The amount of NO was calculated according to the standard curve (top left corner). NIR light (980 nm, 1.5 W/cm^2^) was switched on/off every minute. (**H**) NO release curves of UCZN at different concentrations upon NIR light stimulation (980 nm, 1.5 W/cm^2^) at room temperature during 1 week. The solid lines depict the rapid release of a large amount of NO within 1 day after activation by NIR light. The dotted lines indicate the continuous slow release of NO over the remaining 6 days.

A schematic illustration of UCZN’s synthesis is shown in Fig. 2A, and the corresponding product in each step is shown in Fig. 2B. NaYF_4_: Yb, Tm NPs were first synthesized via a previously validated pyrolysis method (*21*) and then coated by polyvinyl pyrrolidone (PVP) for hydrophilic modification (UCNPs@PVP). This was confirmed by Fourier transform infrared (FTIR) spectra, showing characteristic peaks of PVP at 1291 cm^−1^ (─C─N─) and 1661 cm^−1^ (─C═O) [fig. S1A (a)] (*22*). The prepared UCNPs@PVP NPs were dispersed in methanol containing 2-methylimidazole (2-MI) and Zn^2+^ to form a ZIF-8 layer outside (UCNPs@ZIF-8, abbreviated UCZs) via an epitaxial growth process. ZIF-8 with large pore size and pore surface area in flexible structure can achieve effective drug encapsulation and high drug loading (*23*). The analysis results of FTIR spectra [fig. S1A (b)] and powder x-ray diffraction (XRD; Fig. 2C and fig. S1B) proved the successful preparation of UCZs (*18*). CysNO as the photochemically triggered NO donor was acquired via the nitrosation reaction between L-cysteine (L-Cys) and tert-butyl nitrite (*24*) and was then loaded into the micropores of the ZIF-8 layer in UCZs via physical adsorption, confirmed by the FTIR spectra with new emerging characteristic peaks appearing at 764 cm^−1^ (─S─N═) and 1505 cm^−1^ (─N═O) in UCZN (Fig. 2D) (*25*). Thermogravimetric analysis gave the loading capacity of Cys-SNO in UCZs at approximately 0.35 g of CysNO per gram of UCZN (Fig. 2E). The change of size distribution and zeta potential further certified the successful modification of materials in each step (fig. S1, C and D).

Upon 980-nm laser irradiation, the upconversion emissions of UCNPs (fig. S1E) overlapped with the characteristic absorption of the -SNO group between 330 and 360 nm (n_0_→π* transition) (fig. S1F) (*21*, *26*), allowing efficient energy transfer from the inner UCNPs to the loaded CysNO in UCZN (Fig. 2F). The photoactivity of CysNO comes from the -SNO group, which can release NO via homolytic cleavage of S─N bond (*24*). The dissociation energy of S─N bond is approximately 125 kJ mol^−1^, as given by experimental and theoretical studies (*27*). Moreover, the upconversion fluorescence energy of UCNPs in the range of 330 to 360 nm is around 332 to 361 kJ mol^−1^, calculated using the light quantum energy formula (*E* = *hc*/λ). Therefore, it is expected that the transferred energy from UCNPs to CysNO would cleave the S─NO bond for NO release.

To test this assumption, the Griess photometric method was used to assess the generation of NO from UCZN in aqueous solution. As shown in Fig. 2G, the levels of NO increased substantially after 980-nm laser irradiation for 1 min and then remained almost constant when 980-nm laser was turned off. To simulate NO release in vivo, the solution with UCZN was kept at 37°C and exposed to NIR light on the first day (1.5 W/cm^2^ for 1 min), and the levels of NO were recorded over the next 7 days. Rapid release of NO occurred only after NIR light irradiation, accompanied by slow increase of NO levels in the following days. Notably, the total releasing amount of NO was positively correlated with the concentration of UCZN and number of days, suggesting a dose- and time-dependent effect (Fig. 2H). These characteristics are important to on-demand NO biomolecule NIR photorelease for neuroprotection and neuroregeneration in vivo.

### Cytocompatibility and spatiotemporally controlled NO release of UCZN

Neuron-like PC12 cells were chosen for the evaluation of UCZN’s cytocompatibility and NIR-triggered NO release in vitro. After coculturing with PC12 cells for 4 days, UCZN adhered to the neuronal membrane and located at somata and neurites, as shown by the confocal laser scanning microscopy (CLSM) image with the upconversion luminescence of UCNPs surrounding the green PC12 cells stained with calcein-AM (fig. S2A and movies S1). Cell viability, tested by a typical cell counting kit-8 (CCK-8) assay, was kept above 93% even at a concentration of 160 μg ml^−1^ for up to 7 days, showing the excellent cytocompatibility of UCZN. However, increased levels of cell death were detected when combined with 980-nm laser stimulation, mainly due to a sharp rise of NO release from UCZN (fig. S2B). To determine the safety concentrations of UCZN upon 980-nm laser irradiation for further use, a Live/Dead viability/cytotoxicity assay [calcein-AM/propidium iodide (PI)], an assay of early apoptotic phenomena [annexin V–fluorescein isothiocyanate (FITC)/PI], and a reactive oxygen species (ROS) assay [2,7-dichlorofluorescein diacetate (DCFH-DA)] were implemented, showing no detectable apoptotic response or noticeable oxidative stress when UCZN was at a concentration of no more than 80 μg ml^−1^ (fig. S2C).

**Fig. 3:**
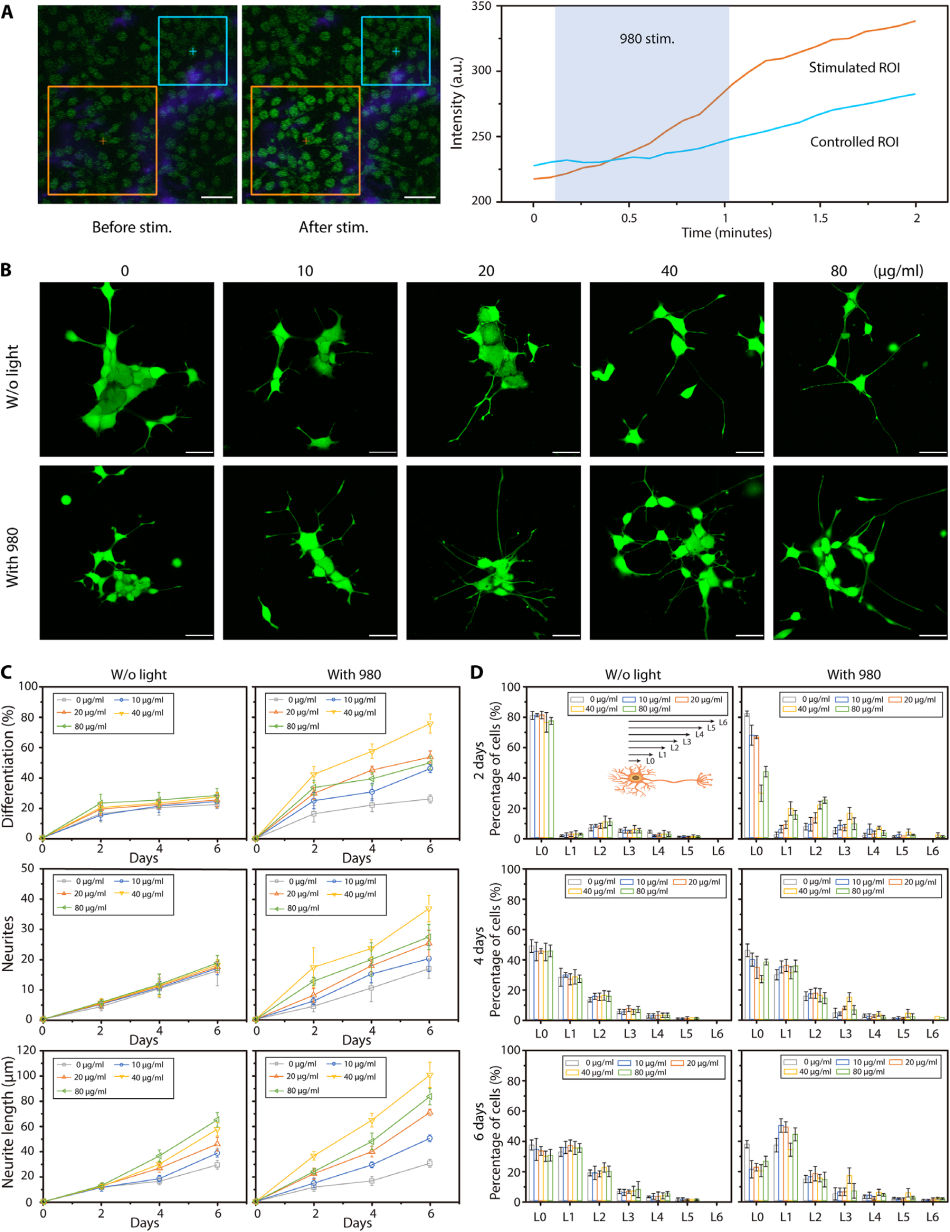
Spatiotemporally controlled release of NO and growth promotion in PC12 cells. (**A**) Confocal microscopy images of PC12 cells precultured with UCZN before and after NIR stimulation. PC12 cells were stained with NO green fluorescence probe (DAF-FM DA). UCZN converted the 980-nm light to blue-violet light. The orange square is the stimulated ROI by NIR light (980 nm, 1.5 W/cm^2^), and the blue square is the control ROI without 980-nm laser stimulation. The real-time curves of signal intensity in different ROIs were shown on the right (scale bar, 50 μm). (**B**) Confocal microscopy images of calcein-labeled PC12 cells after different treatments (varied concentrations of UCZN with or without NIR light) for 6 days (scale bar, 50 μm). (**C** and **D**) Trends of PC12 cell differentiation (*n* = 20, groups = 5, mean ± SD) and the neurite number (*n* = 5, groups = 6, mean ± SD), neurite length (*n* = 6, group = 1, mean ± SD), and growth ratio (*n* = 100, groups = 3, mean ± SD) of PC12 cells over 6 days. The level of growth was quantitatively divided into L0 to L6 according to the length of the neurites, and details are in Materials and Methods.

To verify the ability of UCZN for NIR-triggered NO release in living cells, NO fluorescence probe 3-Amino,4-aminomethyl-2’,7’-difluorescein diacetate (DAF-FM DA) was further used to stain PC12 cells incubated with UCZN (80 μg ml^−1^). When 980-nm laser was locally irradiated in the ROIs (regions of interests) containing UCZN, the intensity of green fluorescence in PC12 cells increased immediately (Fig. 3A and movies S2). By contrast, there was no clear change of green fluorescent signal in the surrounding regions without 980-nm laser stimulation. Notably, NIR stimulation alone has no effect on intracellular NO levels, as shown by the unchanged fluorescent signal in PC12 cells without UCZN pretreatment (fig. S3). Overall, for UCZN, the inner UCNPs played as versatile NIR-optical transducers to generate in situ high-intensity blue-light source, achieving highly spatiotemporal NO release from CysNO for precise therapeutic application in vivo, without systemic adverse effects.

### Neurite growth–promoting property of UCZN

To test the capability of UCZN in nerve repair, PC12 cells, which can differentiate into sympathetic ganglion-like cells by using nerve growth factor (NGF) (*28*), were incubated to investigate the effects of UCZN on cell growth and differentiation. Differentiating rate (the percentage of differentiated cells in the culture), number of neonatal neurites, and neurite length per differentiated cell were monitored every 2 days, and differentiated cells were cocultured with UCZN (0, 10, 20, 40, and 80 μg ml^−1^), irradiated or nonirradiated with 980-nm light (fig. S4A). Figure 3B shows representation images performed on all the groups at the end point.

A substantial increase in the cellular differentiation rate was observed in the groups treated with UCZN and 980-nm light stimulation compared with the UCZN control group. With increasing concentrations of UCZN, the differentiation rates rose from 30 to 80% in the range of 0 to 40 μg ml^−1^ and then fell to 50% at 80 μg ml^−1^ after 980-nm light stimulation (Fig. 3C). Typically, the average number of neonatal neurites per differentiated cell became apparently higher in the culture that underwent both UCZN incubation (40 μg ml^−1^) and 980-nm laser stimulation, showing nearly twice the UCZN control groups. A marked increment of neurite length was also observed, especially at the second day after 980-nm light stimulation ascribing to the NIR-activated rapid NO release in cells (fig. S4B and movies S3).

To quantify the elongation of neural processes, we divided differentiated PC12 cells into seven groups (from L0 to L6) according to the ratio of neurite length to the corresponding length of cell body (Fig. 3D). There was an obvious increase in neurite extension even within 2 days of treatment of UCZN and 980-nm light stimulation, but not in UCZN control groups. At the end of the sixth day, the percentage of L0 in UCZN control groups was 40%, while the percentage decreased to almost 20% in groups of UCZN and NIR stimulation, with increased percentage of high differentiated cells (L3 to L6). In addition, in groups of UCZN and NIR stimulation, the ratio of differentiated PC12 cells showed a dose-dependent raise in the range of 0 to 40 μg ml^−1^ and then decreased when the concentration of UCZN was 80 μg ml^−1^. Together, these observations strongly support the involvement of NO in the neurite outgrowth, and the concentration of UCZN at about 40 μg ml^−1^ is eligible for the development of PC12 neurites and will be used in subsequent experiments.

Encouraged by the above enhancement of neuron development and outgrowth, the effects of UCZN and NIR light on the repair of the injured neurons were further studied. DRG neurons isolated from Sprague-Dawley rats were cultured with UCZN (40 μg ml^−1^) in vitro and then stained with calcein-AM for CLSM imaging of their somata and axons. Calcein-AM is a hydrophobic nonfluorescent probe that can enter into cells and can be hydrolyzed to calcein, an extremely green fluorescent molecule. To construct nerve injury model, the main axons of DRG neurons were locally irradiated by a high-power 405-nm laser (100 mW), causing an acute thermal damage, as proved by the instantaneous disappearance of calcein signal in the stimulated region and the subsequent fall of fluorescence intensity in whole neurons due to the leakage of calcein (fig. S5A and movies S4). Typically, when exposed to 980-nm laser irradiation, the broken regions in the injured axons gradually regenerated as shown by the increased intracellular calcein signal, and after about 4 hours, axon terminals could be seen again. By contrast, these reparative effects were not observed in the NIR light single treatment group (fig. S5B). These results indicated that UCZN under NIR irradiation could even repair the injured neurons, proving hope for SCI treatment in vivo.

### The mechanism of neuromodulation

Rare earth (RE) ions have been reported to promote cell differentiation via the extracellular signal–regulated kinase (ERK) pathway and neurite outgrowth (*29*). However, in our working conditions, the released RE ions from UCZN under NIR light irradiation were too few to work on neuromodulation (table S1).

To determine the role of NO in cell differentiation and growth, the NGF-specific receptor TrkA in PC12 cells was first blocked by K252a (*30*). Then, cultures with different treatments [UCZN (0 or 40 μg ml^−1^) with or without NIR light stimulation] were monitored for 6 days (fig. S6). Differentiation was generally low in all groups, but increased substantially in UCZN with NIR light (*P* < 0.001). Similarly, the number of neurites in the UCZN with the NIR group was twice that in the other groups (*P* < 0.01). Again, a remarkable neurite extension was observed in UCZN with the NIR group, whose neurite length was about 30 μm with respect to below 20 μm of the other groups (*P* < 0.001). In any case, the promoting property of UCZN with NIR light was obviously higher than that of UCZN treated alone (*P* < 0.01), while NIR stimulation alone performed no effect compared to the untreated group. This slight but notable promotion for cell differentiation, even in the presence of an NGF receptor inhibitor, indicated that the NIR-triggered NO release from UCZN can activate signaling molecules of the differentiation pathway, located downstream to the TrkA receptor. Moreover, the increased promoting capacity under NO stimulation without TrkA inhibitor suggests a synergic effect of NGF for NO-induced cell differentiation.

**Fig. 4:**
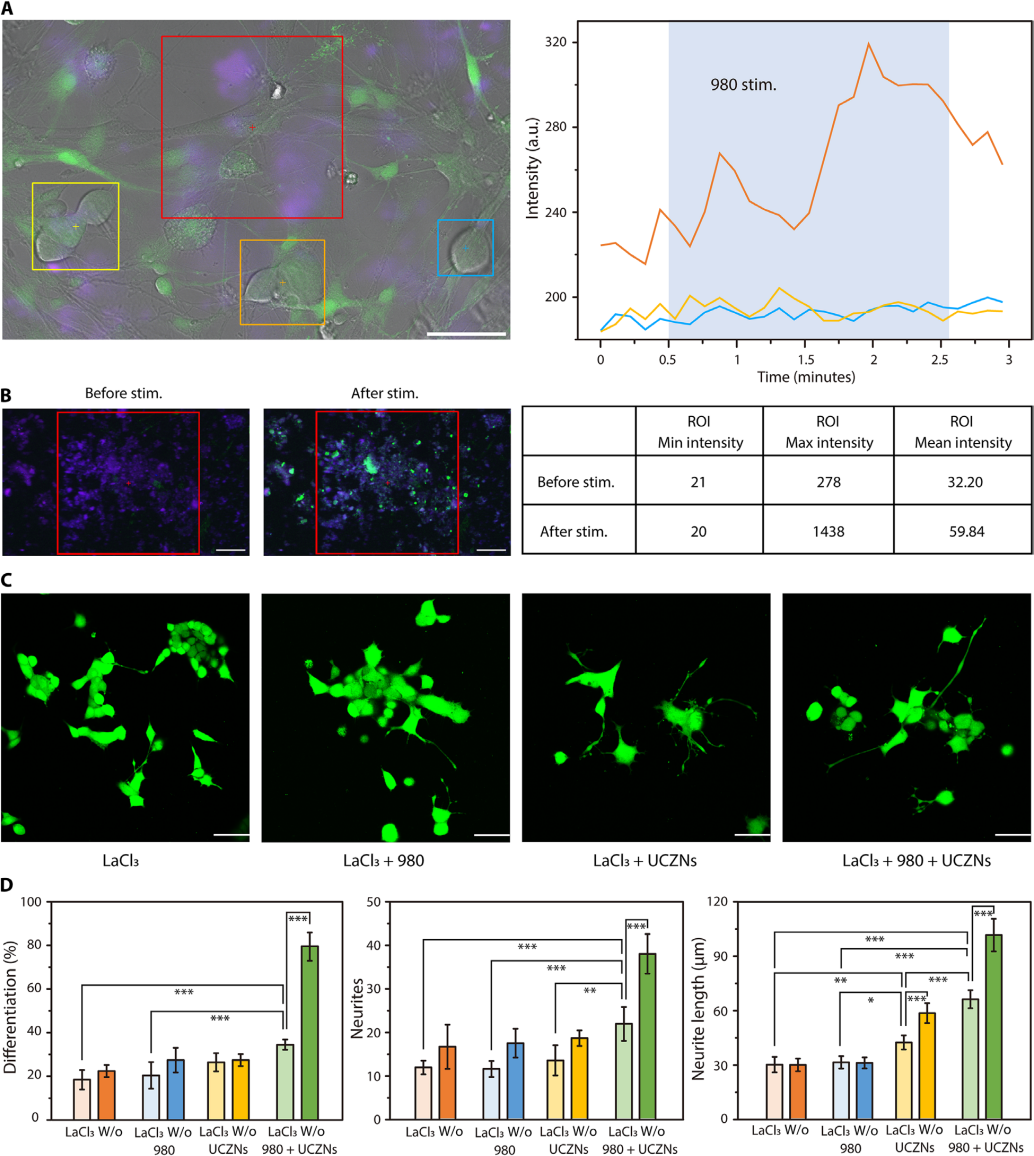
Mechanism of neuronal modulation via UCZN. (**A**) DRG neurons precultured with UCZN (blue-violet) were stained with [Ca^2+^]_in_ green fluorescence probe (Fluo-4 AM). The red square was set as the UCZN-activated ROI with 980-nm laser. Other ROIs (yellow, orange, and blue squares) were selected from DRG neurons, and real-time curves of signal intensity were shown on the right (scale bar, 50 μm). (**B**) [Ca^2+^]_in_ imaging of DRG neurons transfected with GCaMP-X and cocultured with UCZN before and after NIR stimulation. The red square is the stimulated ROI with 980-nm laser irradiation (1.5 W/cm^2^). The real-time change of fluorescence intensity in the selected ROI is shown on the right (scale bar, 200 μm). (**C**) Differentiation and neurite growth status of PC12 cells in the presence of nonspecific calcium ion channel blocker LaCl_3_ under different treatments (scale bar, 50 μm). (**D**) Relevant statistics of cell differentiation (*n* = 20, groups = 5, mean ± SD), neurite number (*n* = 5, groups = 6, mean ± SD), and neurite length (*n* = 6, group = 1, mean ± SD) in comparison with the cases without LaCl_3_. *P < 0.05, **P < 0.01, and ***P < 0.001.

Focusing on NO, it is an important signaling molecule in living body. Studies have suggested that NO can activate cyclic 3′,5′-guanosine monophosphate (cGMP)/protein kinase G (PKG)–dependent phosphorylation, which is important for neurite growth and synapse remodeling after nerve crush (*12*). The cGMP-dependent mechanisms appear to directly or indirectly promote Ca^2+^ mobilization to regulate intracellular Ca^2+^ levels ([Ca^2+^]_in_) (*31*). Calcium signaling also plays a vital role in cell growth and neuronal signal transduction (*32*). To evaluate the effect of NO on [Ca^2+^]_in_, several experiments were carried out. The fluorescent [Ca^2+^]_in_ detection probe (Fluo-4 AM) was used to stain DRG neurons incubated with UCZN (40 μg ml^−1^). Upon localized 980-nm irradiation on UCZN (red square), DRG neurons around UCZN (shown in orange) exhibited instant and marked enhancement of calcium waves, but not in cells far away from UCZN (shown in yellow or blue) (Fig. 4A and movies S5). Moreover, there was no substantial change of [Ca^2+^]_in_ under stimulation with the 980-nm laser alone (fig. S7, A and B). Similar tests were processed on DRG neurons transfected with GCaMP-X for more accurate indication of [Ca^2+^]_in_. After NIR stimulation on UCZN, the localized DRG neurons momentarily fluoresced (Fig. 4B), meaning that the NIR-triggered NO release from UCZN can lead to the sharp rise of intracellular Ca^2+^ levels. Furthermore, the elevated [Ca^2+^]_in_ can activate calmodulin (CaM) to form Ca^2+^-CaM, and so can up-regulate the expression of [Ca^2+^]_in_-related proteins. Western blotting analysis did show the notable expression of CaM and Ca^2+^-calmodulin kinase II (CaMKII) in the UCZN group with 980-nm laser irradiation, further confirming the key role of NO on Ca^2+^ influx in neurons (fig. S7C).

To evaluate the importance of calcium influx, the differentiation and growth of PC12 cells were observed after 6 days of treatment with LaCl_3_, which is known to be a nonspecific calcium ion channel blocker (Fig. 4C) (*33*). In this case, although differentiation was observed in all treatment groups, the promotion of neural process by UCZN and NIR light was again highlighted, with substantial differences in the differentiation rate, the number of developed neurites, and the increment of neurite length (*P* < 0.001). However, there was still a distinct reduction in all measures relative to those obtained in the absence of LaCl_3_ (Fig. 4D). These results suggested that calcium influx caused by NO plays a critical but not the only role in the development of PC12 neurites.

By the very nature of UCZN, NIR-induced NO release from UCZN can regulate [Ca^2+^]_in_ and further promote cellular outgrowth, and this promotion can be more robust in combination with NGF. This is beneficial for repairing the injured spinal cord, because endogenous NGF and TrkA receptor expression increases when the spinal cord is damaged (*34*).

### Repair of SCI in zebrafish and Sprague-Dawley rats

**Fig. 5:**
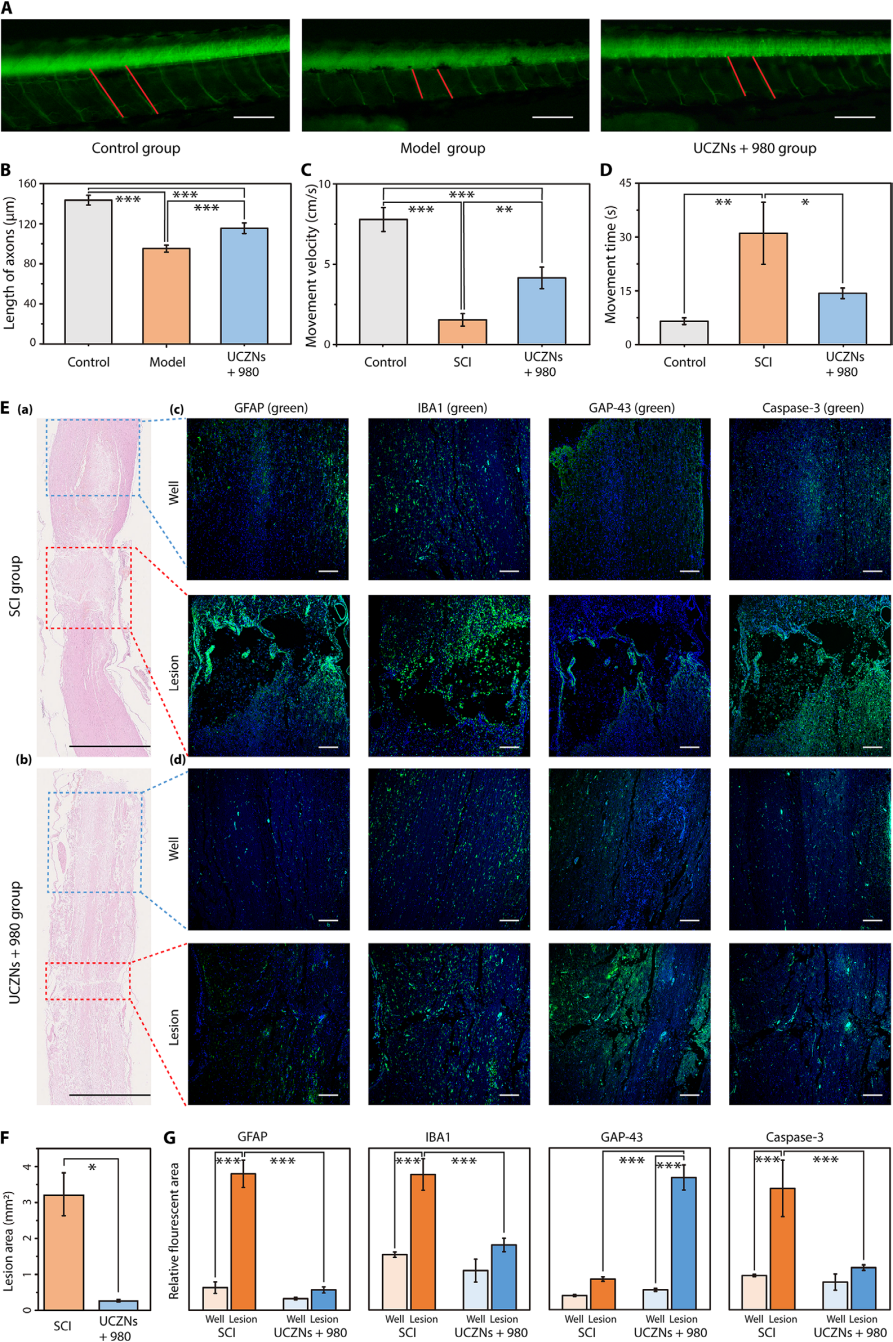
UCZN repaired the injured spinal cord in zebrafish and Sprague-Dawley rats. (**A**) Fluorescent images of the spinal cord of zebrafish under different treatments (scale bar, 100 μm). The motor neuron axons are marked by red lines. (**B**) Average axon length. (**C** and **D**) Movement velocity and time of Sprague-Dawley rats in different groups (*n* = 3, mean ± SD). (**E**) H&E-stained sections of injured spinal cords without (a) or with (b) the UCNP and 980-nm treatment (scale bar, 2500 μm). Representative immunofluorescence images of GFAP, IBA1, GAP-43, and caspase-3 at both the lesion and the well region: without (c) or with (d) UCNPs +980 treatment (scale bar, 200 μm). Cell nuclei were stained with DAPI (4′,6-diamidino-2-phenylindole) in blue. The lesion and the well region were labeled in the H&E-stained sections with red and blue rectangles, respectively. (**F**) The lesion areas were analyzed based on H&E-stained sections (*n* = 3, mean ± SD). (**G**) Relative fluorescent areas of GFAP, IBA1, GAP-43, and caspase-3 were analyzed based on corresponding immunofluorescence images (*n* = 3, mean ± SD). *P < 0.05, **P < 0.01, and ***P < 0.001.

To verify the capability of UCZN in vivo, zebrafish were first used as a model organism in SCI. Randomly selected transgenic zebrafish with fluorescent motor neurons were treated with 1% absolute ethanol to damaged motor neuron axons. Typical micrographs under different treatment conditions are shown in Fig. 5A. One day later, the average length of motor neuron axons in the model group (94 μm) was substantially decreased compared to that in the control group (142 μm, *P* < 0.001), indicating the success of model establishment. After NO delivery activated by NIR light, a distinct increase in the length of motor neuron axons was observed (approximately 42%, *P* < 0.001) in the model group (Fig. 5B and table S2). Meanwhile, the administration of UCZN did not cause other toxic side effects or death in the zebrafish. These results provide evidence to support the positive role of NIR-triggered NO release in the repair of SCI.

Next, the reparative effects of UCZN were further investigated in the spinal cord of mammals. A traumatic SCI model was successfully established by the clip-compression method in adult Sprague-Dawley rats (*35*), characterized by completely paralyzed hindlimbs and limited movement. In the UCZN +980 group, UCZN was locally administered in the regions of SCI and then irradiated with 980-nm laser for 1 min. Four weeks later, the movement velocity and stay time of rats along a walkway of limited length were recorded using a gait analysis system. In contrast to the control group, the movement velocities in both the SCI group without any treatment and the UCZN +980 group were very low, but the velocity in the UCZN +980 group was markedly higher (greater than 4 cm/s) than that in the SCI group (approximately 1 cm/s) (*P* = 0.005) (Fig. 5C). Meanwhile, the rats with SCI with or without UCZN +980 treatment all tended to take longer time to complete the process due to stopover when compared to that in control. However, the stay time in the UCZN +980 group was remarkably lower than that in the SCI group (*P* = 0.016). Notably, there was no notable difference in motor performance between the UCZN +980 group and the control group (Fig. 5D). Together, these observations strongly support the key role of NO in SCI repair for improved motor ability and function of the SCI rats.

After the behavioral experiments, the whole spinal cords were dissected out for histological analyses. The lesion area was assessed in hematoxylin and eosin (H&E)–stained longitudinal spinal cord sections [Fig. 5E (a and b)]. A substantial decrease of the traumatic lesion area was found in the UCZN +980 group compared to the control SCI group (about 90%, *P* = 0.013) (Fig. 5F), again confirming the effects of NO on SCI repair. To explore the underlying mechanism, the inflammatory reaction of SCI, and neuronal growth and apoptosis in the lesion regions were studied via immunofluorescence staining. Usually, the inflammatory response to SCI can activate the expression of inflammatory factors and can also bring an increased number of astrocytes and microglia with high activation (*5*). Therefore, the expression of three primary inflammatory factors [interleukin-1 (IL-1), IL-6, and tumor necrosis factor–α (TNF-α)], GFAP [glial fibrillary acidic protein; a marker of astrocyte activation (*36*)], and IBA1 [ionized calcium binding adapter molecule 1; a microglia-specific protein (*37*)] was detected to evaluate the degree of inflammatory reaction. Besides, growth associated protein-43 (GAP-43) as a neural-specific protein was expressed at high levels during the development and regeneration of neurons (*4*, *38*). Thus, GAP-43 as an index was tested to assess the growth of neurons. Caspase-3, which is the most critical executioner of apoptosis, was also detected for apoptosis analysis (*39*). The immunoreactivity to each marker at both the lesion site of SCI and the well spinal cord around was recorded as shown in Fig. 5E (c and d) and fig. S8. The immunoreactivity of IL-1, IL-6, TNF-α, GFAP, IBA1, and caspase-3 at the lesion area was substantially higher than that at the well spinal cord in the SCI group (*P* < 0.01 for inflammatory factors and *P* < 0.001 for GFAP, IBA1, and caspase-3), while this difference disappeared in the UCZN +980 treatment group, with all regions showing low immunoreactivity to inflammatory factors—GFAP, IBA1, and caspase-3—meaning that NO can effectively inhibit gliosis, inflammatory response, and apoptosis for neuroprotection. Meanwhile, the GAP-43 immunoreactivity in the lesion area increased substantially (*P* < 0.001) in the treatment group than in the SCI group, suggesting a positive impact on neuroregeneration (Fig. 5G). Generally, the immunohistochemical and immunofluorescent results further confirmed the promoting effect of UCZN on nerve regeneration, which was consistent with previous experimental results.

## DISCUSSION

In this study, we constructed a NIR-triggered NO release nanosystem with dual role in neurogeneration and neuroprotection for effective repair of traumatic SCI. Upon NIR light stimulation, the released NO rapidly reached a physiological level, leading to the activation of the differentiation pathway for a markedly pronounced outgrowth of neuronal processes in both PC12 cells and DRG neurons. In vivo experiments showed that UCZN, after one 980-nm laser irradiation, substantially promoted the outgrowth of the damaged motor neuron axons in zebrafish, as well as facilitated the motor rehabilitation in Sprague-Dawley rats with traumatic SCI, ascribable to the pleiotropic effects of NO including the suppression of gliosis and inflammation, the promotion of neuroregeneration, and the protection of neurons from apoptosis. Our results, collectively, open intriguing perspectives not only in nerve repair but also in neurological research and tissue engineering.

## MATERIALS AND METHODS

### Materials

YCl_3_·6H_2_O, YbCl_3_·6H_2_O, TmCl_3_·6H_2_O, and 1-octadecene (90%) were procured from Shanghai Macklin Biochemical Co. Ltd. Oleic acid was purchased from Sigma-Aldrich. PVP [molecular weight (*M*_w_) = 40,000] was obtained from Aladdin. Zn(NO_3_)_2_·6H_2_O, sodium hydroxide (NaOH), and trichloromethane were purchased from Sinopharm Chemical Reagent Co. Ltd. 2-MI and LaCl_3_ were obtained from Adamas-beta. L-Cys and tert-butyl nitrite were purchased from Tokyo Chemical Industry (TCI). Ammonium fluoride (NH_4_F), sodium nitrite (NaNO_2_), absolute methanol, and absolute ethanol were obtained from General Reagents. All reagents were of analytical grade and used directly without further purification. DAF-FM DA, DCFH-DA, CCK-8, Annexin V-FITC Apoptosis Detection Kit (annexin V–FITC/PI), Hoechst 33342, and Fluo-4 AM were purchased from Beyotime Biotechnology. The Calcein-AM/PI Double Stain Kit was obtained from Yeasen. K252a was purchased from Alomone.

### Characterization

Transmission electron microscopy (TEM) images were obtained using a JEOL electron microscope at 200 kV (JEM-2100F, Japan). XRD patterns were acquired on a Rigaku D/MAX-2250V diffractometer. The hydrodynamic radius and zeta potential were collected via dynamic light scattering (DLS) using Zetasizer Nano-ZS (Malvern, UK). UV-visible (UV-Vis) absorption spectroscopy was measured on a Shimadzu UV-3600 Plus spectrophotometer. FTIR spectra were obtained with a Bruker TENSOR II FTIR spectrometer. Fluorescence analysis was measured on an Edinburgh FLS980 spectrometer. Thermogravimetric analysis was conducted with a Netzsch STA449F3 synchronized thermal analyzer (DSC/DTA-TG). RE ion concentrations were recorded with an Agilent 5110 ICP-OES (inductivity coupled plasma optical emission spectrometer). The amount of NO was detected on a Spark multimode microplate reader (Tecan, Switzerland). CCK-8 assays were conducted with a Bio-Tek ELx800 microplate reader and analyzed with Gen5TM data analysis software. CLSM images were obtained using a Nikon A1+R-980 confocal microscope (Japan). Fluorescence microscope images were captured by Nikon AZ100 (Japan).

### Synthesis of UCNPs

The UCNPs were prepared following the method reported in our previous paper. The NaYF_4_: Yb(20%), Tm(0.2%) NPs were synthesized by a pyrolysis process. Briefly, YCl_3_·6H_2_O (485.28 mg, 1.6 mmol), YbCl_3_·6H_2_O (155.00 mg, 0.4 mmol), and 5 μl of 0.8 M TmCl_3_·6H_2_O aqueous solution were added to a 100-ml flask containing 15 ml of oleic acid and 30 ml of 1-octadecene (90%). The mixed solution was heated to 120°C in the absence of O_2_ under stirring and then kept at this temperature for 1 hour. After cooling down to room temperature, a 10-ml methanol solution of NaOH (200 mg, 5 mmol) and NH_4_F (296.3 mg, 8 mmol) was added to this system for 2 hours. Then, the temperature of solution was raised to 110°C to adequately remove methanol and water. The mixture was rapidly heated to 290°C and maintained for 1.5 hours and then cooled down to room temperature. The resulting NaYF_4_: Yb, Tm NPs were washed with ethanol and cyclohexane for several times and dispersed in 4 ml of cyclohexane.

### Synthesis of UCZs

The methods of PVP modification and ZIF-8 coating were described in previous work. Two milliliters of UCNPs was dissolved in 10 ml of trichloromethane and mixed with 15 ml of trichloromethane of 1.67 mM PVP (1 g, *M*_w_ = 40,000) in a 100-ml flask. The mixture was stirred for 24 hours at 40°C to modify PVP on UCNPs. After removing trichloromethane by ethanol washing, UCNPs@PVP was dispersed in 10 ml of absolute methanol. Then, 2 ml of UCNPs@PVP solution, 10 ml of 2-MI (25 mM), and 10 ml of Zn(NO_3_)_2_·6H_2_O (25 mM) were mixed and then allowed to react at room temperature for 24 hours under gently stirring. The products were collected by centrifugation, washed several times with ethanol, and finally dissolved in 2 ml of absolute ethanol.

### Synthesis of UCZN

L-Cys (0.6 mmol) and tert-butyl nitrite (100 μl) were mixed in 2 ml of absolute ethanol under alkaline condition to generate RSNOs. Then, 2 ml of the prepared UCZs was added to this solution under stirring for 4 hours, and CysNOs were loaded in ZIF-8 shell. The resulting UCZN was washed with ethanol several times, rapidly dried in vacuum, and stored at dark conditions.

### Measurement of NO release in deionized water

NO release from UCZN upon NIR irradiation was quantitatively measured using a typical Griess assay. When in contact with water, the released NO could be converted into nitrite (NO_2_^−^). After reaction with the Griess agent, the nitrite was finally transformed into an azo dye that could be quantitatively determined using a microplate reader (Tecan, Switzerland) at λ = 540 nm. A standard curve between the absorbance intensity and the nitrite concentration was made by NaNO_2_ with known concentrations. The background absorbance at λ = 540 nm caused by UCZs was deducted.

### Cell culture

PC12 cell lines derived from transplantable male rat adrenal pheochromocytoma were purchased from the Cell Bank of Type Culture Collection of the Shanghai Institute of Cell Biology, Chinese Academy of Sciences, where they passed mycoplasma detection. Cells were used without modification. They were incubated in RPMI 1640 (Gibco) medium supplemented with 5% fetal bovine serum (FBS; Gibco), 10% horse serum (Gibco), 1% penicillin/streptomycin (Gibco), and 2 mM l-glutamine (Adamas) for proliferation or supplemented with 2% FBS, 1% penicillin/streptomycin, 2 mM l-glutamine, and NGF (50 ng ml^−1^) for differentiation and growth.

DRG neurons were excised from 4-week-old Sprague-Dawley rats into Dulbecco’s modified Eagle medium (DMEM) medium on ice and then digested in a mixture of deoxyribonuclease I (1 mg ml^−1^), trypsin (4 mg ml^−1^), and collagenase (10 mg ml^−1^) (Sigma-Aldrich) for 60 min at 37°C. The digested DRG neurons were resuspended into DMEM to inhibit further digestion and purified by Percoll (GE). The resulting dispersed DRG neurons were then resuspended into DMEM/F12 (1:1) + 5% FBS + 1% N_2_ + NGF (50 ng ml^−1^) + 20 μM 5-fluorouracil (5-FU) for long-time culture. All these cells were maintained at 37°C with 95% air and 5% CO_2_.

### Cell viability assay

Cell toxicity was tested by a standard CCK-8 assay. Briefly, suspended PC12 cells were seeded in 96-well cell culture plates (10,000 cells per well) and cultured in differentiating medium for 24 hours before treatment with UCZN. Then, UCZN was added into the medium at concentrations ranging from 10 to 160 μg ml^−1^ with or without NIR irradiation for 1 min and maintained for 7 days before discarding the cell supernatant. After washing twice with phosphate-buffered saline (PBS), 100 μl of CCK-8 solution was added to each well for another 2 hours. The absorbance of each well was measured at 450 nm, and the cell survival rate was obtained in relation to that in the control groups.

Viability was further investigated with a Live/Dead viability/cytotoxicity kit. After incubation with different concentrations of UCZN (ranging from 10 to 160 μg ml^−1^) and 1-min NIR irradiation, the cells on day 7 were stained with 100 μl of 2 mM calcein-AM and 1.5 mM PI for 15 min at 37°C in 1× buffer. For early apoptosis and ROS production detection, the cells were treated with UCZN (10 to 160 μg ml^−1^) and 1-min NIR irradiation for 7-day culture and then treated with Hoechst 33342 (5 μg ml^−1^) and annexin V–FITC (20 μg ml^−1^) or 10 μM DCFH-DA for 20 min at 37°C in phenol red–free DMEM. The results of these tests were observed by CLSM.

### Real-time probing of NO release in PC12 cells

The differentiated PC12 cells were cocultured with UCZN (80 μg ml^−1^) and then stained with 5 μM DAF-FM DA for 20 min at 37°C in the dark before the imaging experiments. The NO release was recorded by sequential 980-nm laser (1.5 W/cm^2^) stimulation and 488-nm laser (0.1 mW) imaging under CLSM.

### Measurement of neurite growth on PC12 cells

Neurite growth of PC12 cells was measured in a dose- and time-dependent manner. PC12 cells were cultured in differentiating medium and treated with UCZN of different concentrations (from 0 to 80 μg ml^−1^) with or without NIR irradiation, which was settled at 1.5 W/cm^2^ for 1 min. After differentiation induction at 2, 4, and 6 days, the morphology of PC12 cells was visualized by CLSM. Meanwhile, the differentiation (*n* = 20, groups = 5), neonatal neurites (*n* = 5, groups = 6), neurite length (*n* = 6, group = 1), and growth ratio (*n* = 100, groups = 3) of PC12 cells were analyzed by counting the number of cells and measuring the length of neurites by NIS-Elements. Specifically, 20 cells were randomly selected from the confocal images in different experimental groups, and their differentiation rate was counted. Then, another four groups were repeated, and the mean value and SD between the five groups were calculated. For neurites, five synaptic cells were randomly selected from the confocal images of different experimental groups, the number of synapses in each cell was counted and then repeated five times, and the mean value and SD between the six groups were calculated. For neurite length, six synapses were randomly selected, their lengths were counted, and the mean value and SD between them were calculated. For growth ratio analysis, 100 cells were randomly selected to compare their synapses with their cell size, and the ratio of L0-L6 was calculated under different experimental conditions and repeated two times, and the mean value and SD between the three groups were calculated. In addition, L0 was defined as cells with neurites whose length was shorter than the size of the cell body. L1 was defined as cells with neurites whose length was between the original and twice the size of the cell body. L2 was defined as cells with neurites whose length was between the twice and triple size of the cell body.

For pharmacological inhibition of TrkA or calcium ion channels, cultures were treated with 200 nM K252a or 100 μM LaCl_3_ and then monitored up to 6 days. Four parallel experiments were carried out: cells cultured in differentiating medium with and without NIR light stimulation and cells cultured with UCZN (40 μg ml^−1^) both stimulated and nonstimulated with NIR light. Differentiation, neonatal neurites, and neurite length were also analyzed as above.

### Repair of laser-induced damage on DRG neurons

The purified DRG neurons were incubated in glass bottom culture dish (Nest) and treated with UCZN (40 μg ml^−1^) for one night. Then, the DRG neurons were stained by calcein-AM and monitored via CLSM. With the continuous imaging of 488-nm laser (0.1 mW) for DRG neurons, one of the axon terminals was continuously stimulated by high-power 405-nm laser (100 mW) to induce a damage of axon terminal. Then, the DRG neurons with formed damage on axon were irradiated by 980-nm laser (1.5 W/cm^2^) for 1 min. Living cell culture system was used to monitor in real time the repair of the damaged axon through CLSM for 8 hours.

### Western blot analysis

Protein lysates were extracted from PC12 cells (harvested at the second day after treatment with UCZN and NIR light) by incubating with radioimmunoprecipitation assay (RIPA) lysis and phenylmethylsulfonyl fluoride (PMSF) buffer (Absin). Protein concentration was determined using a BCA protein assay kit (Beyotime Biotechnology). Equal amounts of proteins were separated by SDS–polyacrylamide gel electrophoresis, then transferred to polyvinylidene difluoride membranes (Pall Corp.), and incubated overnight at 4°C with primary antibodies followed by blocking with bovine serum albumin (5%, v/v). The primary antibodies included CaMKII (rabbit, GB11673, 1:500) and calmodulin 1/2/3 (rabbit, ab45689, 1:10,000). The membranes were probed with the indicated secondary antibodies and scanned with Odyssey instruments (LI-COR).

### RE ion release in deionized water

To assess the RE ionic release from the UCZN in an aqueous phase, the comparison of NaYF_4_: Yb, Tm with or without NIR irradiation was used to study the ionic effect. The UCZN in different concentrations was irradiated with NIR for 1 min, and the RE ion concentrations in the dialysate were detected by ICP-OES.

### Recovery of SCI on zebrafish

The transgenic zebrafish with fluorescent motor neurons were bred in a natural paired manner and fed in water for fish farming at 28°C (water quality: 200 mg of instant sea salt per 1 liter of reverse osmosis water; conductivity was 480 to 510 μs/cm; pH was 6.9 to 7.2; hardness was 53.7 to 71.6 mg liter^−1^ CaCO_3_). Feeding management met the requirements of international Association for Assessment and Accreditation of Laboratory Animal Care (AAALAC) certification. All zebrafish experiment operations agreed with the protocols approved by Institutional Animal Care and Use Committee (IACUC) (no. 001458). Randomly selected transgenic zebrafish after fertilization for 6 hours were incubated in a six-well plate with 30 zebrafish per well. The SCI model was established on zebrafish induced by 1% absolute ethanol. After 24 hours of treatment with absolute ethanol, the solution was changed. In another 24 hours, the solution was replaced by UCZN (40 μg ml^−1^) in fish farming water and irradiated for 1 min. The control group (zebrafish only treated with fish farming water) and the model group were set up. Then, the phenotype and mortality of zebrafish were observed and recorded under fluorescence microscope. The axon lengths of two motor neurons were measured and analyzed to evaluate the protective effects of UCZN on SCI in zebrafish. The promoting ratio was calculated as follows:

$$\text{Protective effect}\;(\%)=\frac{S(\text{UCZN}+980\;\text{group})-S(\text{Model group})}{S(\text{Control group})-S(\text{Model group})}\times 100\%$$

### The repair of traumatic SCI on Sprague-Dawley rats

#### Animals and ethics statement

Twenty male Sprague-Dawley rats (180 g) were purchased by Shanghai Laboratory Animal Center, Chinese Academy of Sciences and fed 2 to 3 per cage at room temperature and adequate diet. These rats were randomly divided into three groups (*n* = 6): normal control, SCI, and UCZN +980. All Sprague-Dawley rat experiment operations agreed with the care regulations approved by the administrative committee of laboratory animals of East China Normal University and the protocols approved by IACUC (m^+^R20190701).

#### Clip-compression SCI model

The rats were anesthetized by intraperitoneal injection of 10% chloral hydrate (0.3 ml/100 g). Then, the rats were in prone position on a heat lamp, and dorsal hairs were cut off and the skin was disinfected. A longitudinal incision was made to expose the spinal cord. We produced clip-compression SCI in the SCI and UCZN +980 groups by thoracic laminectomy (Th8-Th9) using aneurysm clamp for 30 s with a closing force of 70*g*. The outer membrane was sutured with 9-0 prolene end to end, and the anastomosis without tension was kept. UCZN was mixed with medical gel and applied around the anastomosis and then irradiated by 980-nm laser for 1 min, and the muscles and skin were sutured layer by layer. After the establishment of the model, the sense and reflex of these rats were lost with a Basso, Beattie, and Bresnahan (BBB) score of <2. The hindlimbs of post-SCI rats did not move autonomously, and their knee joint, ankle joint, and toe joint could not bend, droop or support the body, and rely on the drag of other limbs to move, indicating the success of the model. After operation, each rat was raised in cages, and iodophor was given to the incision every day until healed.

#### Behavior test

Four weeks after operation, the locomotor capacity of rats in normal, SCI, and UCZN +980 groups was performed using a CatWalk XT gait analysis system (version 10.6, Noldus, The Netherlands). These rats stayed in a dark room and walked on a glass platform that was illuminated from the side. The light could record the position of rats’ footprints when they touched the platform. The velocity and time of movement were analyzed.

### Immunohistochemical analysis

Rats in the SCI and UCZN +980 groups were executed at a certain point in time (just after SCI and 4 weeks after injury). The spinal cord was separated and sectioned longitudinally. The sections were baked at 60°C for 1 hour and immersed into xylene and ethanol for dewaxing. Part of the sections was stained in H&E after washing with tap water or PBS. Other sections were immersed in sodium citrate buffer solution (0.01 M, pH 6.0) at high pressure for 5 min to repair the antigen, followed by cooling for 40 min. After inactivating peroxidase (in 3% H_2_O_2_ solution for 15 min), the sections were incubated overnight at 4°C with primary antibodies. The primary antibodies included IL-1 (rabbit, bs-0812R, 1:1000), IL-6 (rabbit, bs-0782R, 1:600), and TNF-α (rabbit, bs-2081R, 1:500). The sections were incubated for 30 min at room temperature with a secondary antibody [goat anti-rabbit immunoglobulin G (IgG), 1:100, Jackson ImmunoResearch Laboratories]. The nuclei were restained by hematoxylin. After that, all slices were immersed into clean xylene transparent for 5 min and sealed by neutral gum. The sections were observed with a 40-fold objective lens to determine the growth status. The lesion areas in the visual field were calculated with ImageJ (National Institutes of Health, USA).

### Immunofluorescence analysis

The spinal cord sections of rats in the SCI and UCZN +980 groups were obtained as previously described. The antibodies used in this study involved GFAP (mouse, MAB3402, 1:1800), IBA1 (rabbit, #17198s, 1:200), GAP-43 (rabbit, bs-0154R, 1:200), and cleaved caspase-3 (rabbit, #9664, 1:30). The secondary antibodies were goat anti-rabbit IgG (1:100, Jackson ImmunoResearch Laboratories) and goat anti-mouse IgG (1:100, Jackson ImmunoResearch Laboratories). The sections were fixed with immunostaining fixative solution. After fixation, the sections were washed twice with immunostaining washing solution for 5 min each time. The immunostaining solution was added and sealed at 4°C for 1 hour. The blocking solution was removed, and the diluted primary antibody was added and incubated at 4°C for 1 hour. The first reactant was recovered and washed with washing solution three times for 5 min each time. The second antibody staining was carried out in a similar way. After staining, fluorescent images of each marker were taken at the same exposure conditions and time. The average fluorescent intensity of each image was analyzed with ImageJ.

### Data analysis

Statistical analysis for two independent samples was done using two-tailed unpaired *t* test with Welch’s correction (Fig. 5F), while statistical analysis for multiple independent samples was achieved by using one-way analysis of variance (ANOVA) (Fig. 5, B to D) or two-way ANOVA (Figs. 4D and 5G), all with Tukey’s multiple comparisons test on GraphPad Prism 8. All data statistics and charts making were obtained by using Microsoft Office Excel 2019 or OriginPro 8. The data layout was designed using Adobe Illustration CC 2018. Data shown are mean ± SD.

## Supplementary Materials

### This PDF file includes:

Supplementary Texts Figs. S1 to S8 Tables S1 and S2 Legend for movies S1 to S5

### Other Supplementary Material for this manuscript includes the following:

Movies S1 to S5

Erratum published 4 July 2025:
The original version of the Research Article contained an error in the Supplementary Materials. The following corrections have been implemented: • In the originally published version of fig. S2C, the image for “Live/dead, 80 μg/mL” was inadvertently a duplicate image of the image for “Live/dead, 10 μg/mL.” This error occurred during the figure assembly process. Figure S2C has been corrected to include the correct image for “Live/dead, 80 μg/mL.” The authors’ conclusions are not affected by these corrections. The Supplementary Materials have been updated. The original version is available here:

View/request a protocol for this paper from *Bio-protocol*.
