# Supplementary material for: Near-infrared light-triggered NO release for spinal cord injury repair
Source: Sci Adv. 2020 Sep 25;6(39):eabc3513. doi: 10.1126/sciadv.abc3513 (PMC7518874; doi:10.1126/sciadv.abc3513)
Supplement: Supplementary file 1 — Supplementary Texts Figs. S1 to S8 Tables S1 and S2 Legend for movies S1 to S5 [file sciadv.abc3513_sm.v2.pdf]

ERRATUM PUBLISHED 4 JULY 2025; SEE BELOW

Science Advances
AAAS

advances.sciencemag.org/cgi/content/full/6/39/eabc3513/DC1

Supplementary Materials for

Near-infrared light-triggered NO release for spinal cord injury repair

Yaqin Jiang, Pengfei Fu, Yanyan Liu*, Chaochao Wang, Peiran Zhao, Xu Chu, Xingwu Jiang, Wei Yang, Yelin Wu, Ya Wang, Guohua Xu*, Jin Hu, Wenbo Bu*

*Corresponding author. Email: wbbu@fudan.edu.cn (W.B.); liuyanyan@chem.ecnu.edu.cn (Y.L.); xuguohuamail@smmu.edu.cn (G.X.)

Published 25 September 2020, *Sci. Adv.* **6**, eabc3513 (2020)
DOI: 10.1126/sciadv.abc3513

The PDF file includes:

Supplementary Texts
Figs. S1 to S8
Tables S1 and S2
Legend for movies S1 to S5

Other Supplementary Material for this manuscript includes the following:

(available at advances.sciencemag.org/cgi/content/full/6/39/eabc3513/DC1)

Movies S1 to S5

Erratum (4 July 2025): There were errors in the originally published version of fig. S2C in the Research Article “Near-infrared light-triggered NO release for spinal cord injury repair” by Y. Jiang *et al.*

The following correction has been implemented:

- In the originally published version of fig. S2C, the image for “Live/dead, 80 μg/mL” was inadvertently a duplicate image of the image for “Live/dead, 10 μg/mL.” This error occurred during the figure assembly process. Figure S2C has been corrected to include the correct image for “Live/dead, 80 μg/mL.”

The authors’ conclusions are not affected by these corrections. The Supplementary Materials have been updated.

Figures and Tables

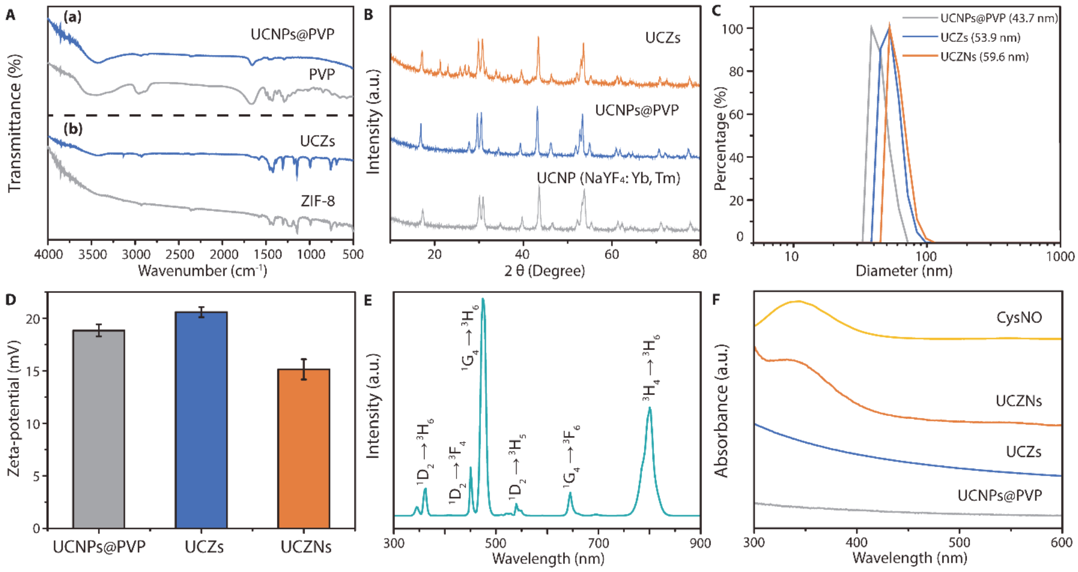

fig. S1. Characterization of UCZNs. (**A**) FTIR spectra of UCNPs@PVP and UCZs in contrast with that of pure PVP and ZIF-8, respectively. (**B**) XRD patterns of samples in every step during the synthesis process. (**C** and **D**) The changes of size distribution and Zeta-potential during the modification process. (**E**) Fluorescence emission spectra of UCNPs under 980 nm light excitation. (**F**) UV/Vis spectra of CysNO and the acquired nanomaterials in each step during the synthesis process.

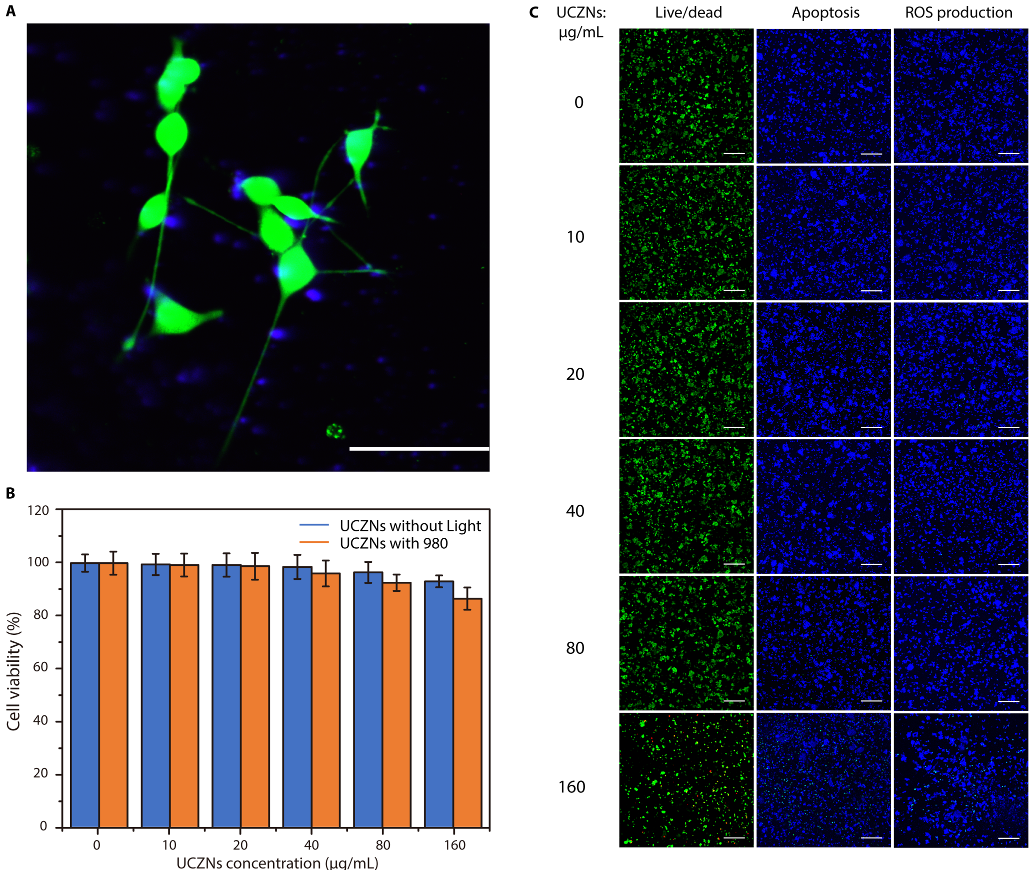

fig. S2. The biocompatibility of UCZNs. (**A**) The co-location of UCZNs and PC12 cells. PC12 cells was labeled by Calcein-AM in green and UCZNs was excited by 980 nm laser emitting blue-violet light (Scale bar, 100 μm). (**B**) The cytotoxicity effects of UCZNs on PC12 cells by CCK-8 assay after 7 days of co-incubation. (**C**) The cytocompatibility of UCZNs on PC12 cells by the live/dead assay (green in live cells and red in dead cells), apoptosis (blue in nuclei and green in apoptotic cells) and ROS detection (blue in nuclei and green represented ROS rose) after 7 days of co-incubation (Scale bar, 200 μm).

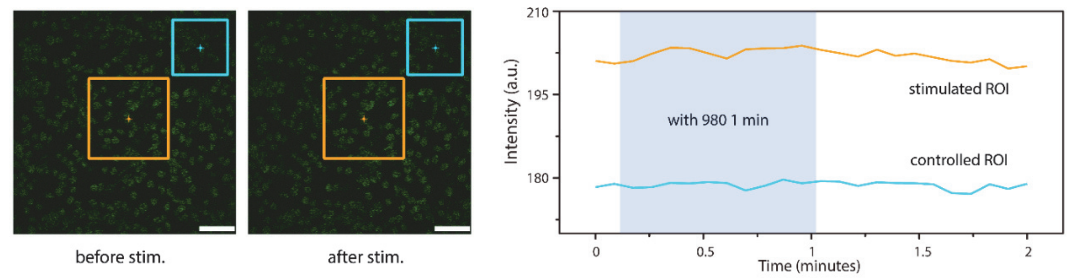

fig. S3. The effect of NIR on NO level in PC12 cells. Real-time recording of NO in PC12 cells without UCZNs under NIR stimulation. PC12 cells were labeled by DAF-FM DA (Scale bar, 50 μm).

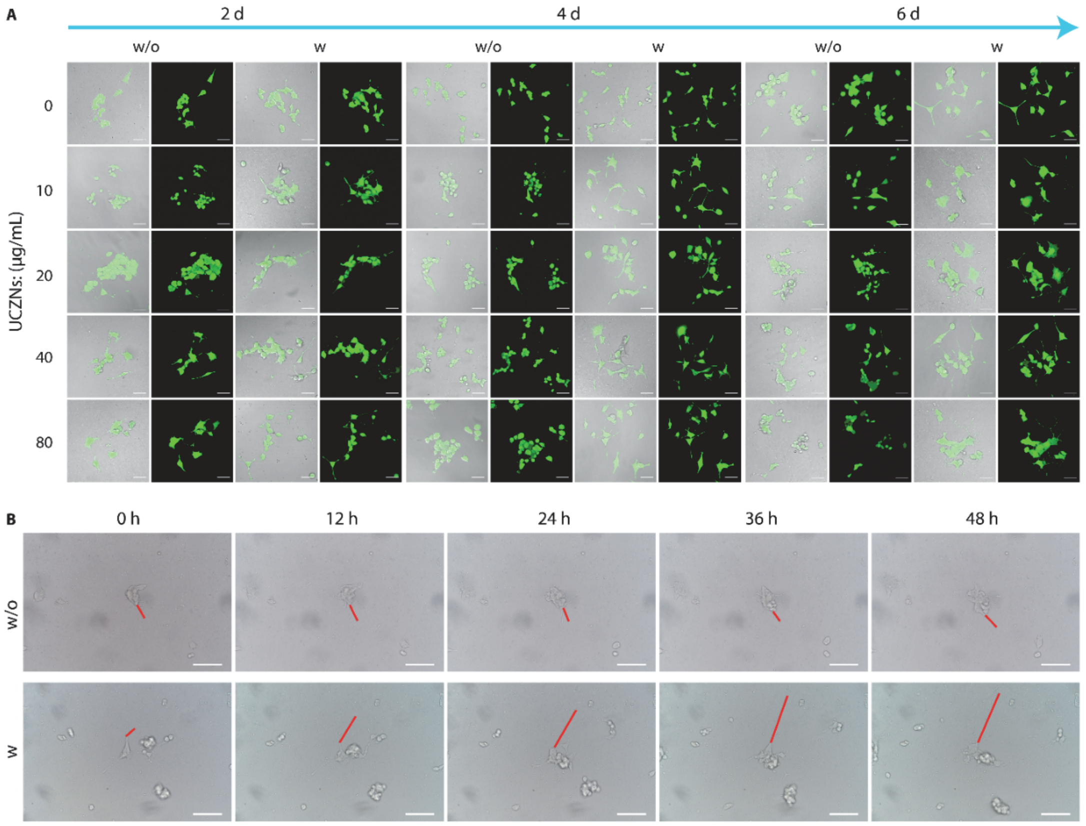

fig. S4. The growth of PC12 cells with different treatments. (**A**) The states of differentiation and neurite growth in PC12 cells under different treatments. The Calcein-AM dye was used for staining PC12 cells (Scale bar, 50 μm). (**B**) The states of differentiation and neurite growth in PC12 cells with 40 μg mL^{-1} UCZNs, with (W) or without (W/O) NIR light irradiation for 48 h real-time monitoring (Scale bar, 50 μm).

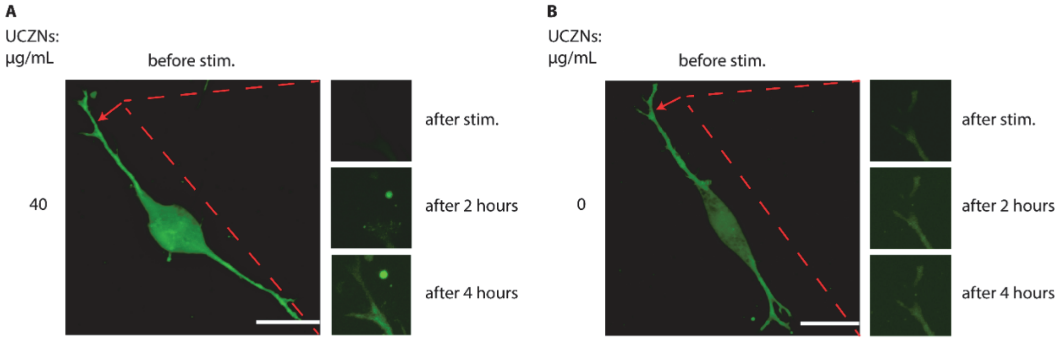

fig. S5. The repair of injured neurons with UCZNs and NIR light. Visualizing the morphological changes in DRG neurons with (**A**) or without (**B**) UCZNs under 980 laser irradiation. The red arrows referred to the damage site caused by high power laser (Scale bar, 25 μm).

UCZNs (μg/mL)	10	20	40	80	160
Y^{3+}	1.61	2.65	2.92	3.31	2.78
Yb^{3+}	0.54	0.95	1.19	2.22	4.17
Er^{3+}	0.01	0.02	0.03	0.05	0.06

table S1. The amount of RE ions release under NIR irradiation from UCZNs.

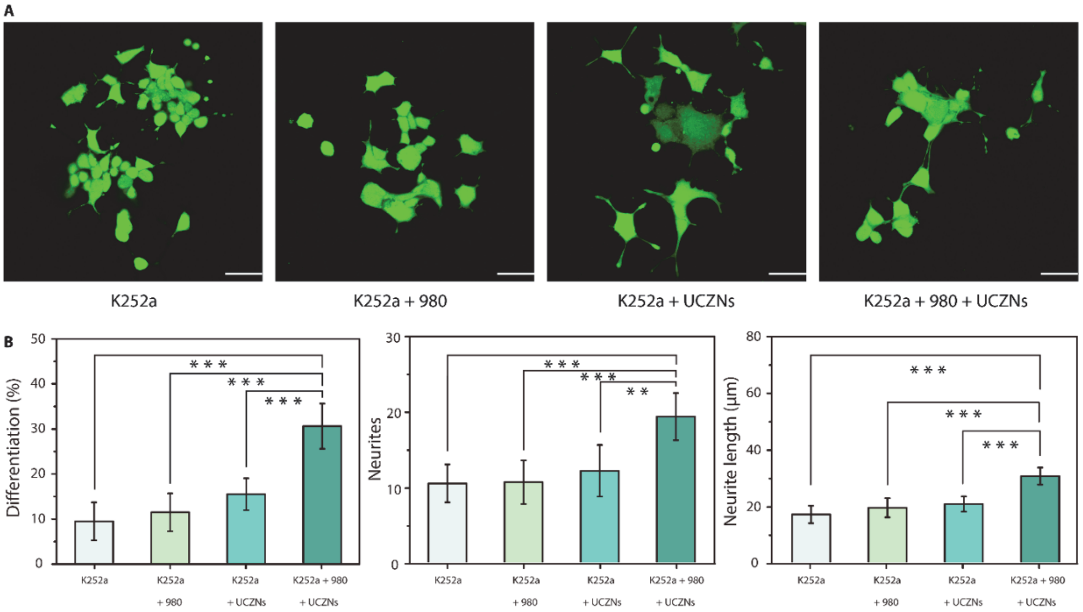

fig. S6. The influence of K252a on the growth of PC12 cells. (**A**) The differentiation of and neurite growth status of PC12 cells in the presence of the nonspecific calcium ion channel blocker $LaCl_3$ with other different treatments (scale bar, 50 μm). (**B**) Trends of PC12 cells differentiation (n = 20, groups = 5, mean ± S.D.), neurite number (n = 5,

groups = 6, mean ± S.D.) and neurite length (n = 6, group = 1, mean ± S.D.) treated with 980 nm laser, UCZNs or both after adding $LaCl_3$. Significance was set at *P < 0.05, **P < 0.01, and ***P < 0.001 analyzed by one-way ANOVA with Tukey's multiple comparison test.

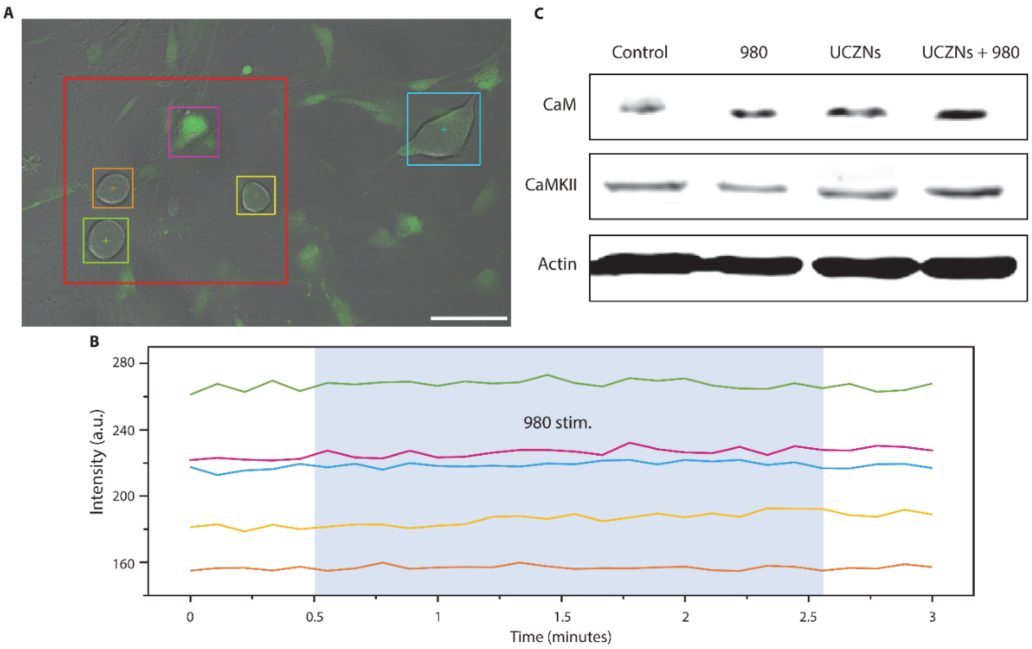

fig. S7. The effect of NIR on $[Ca^{2+}]_{in}$ signal and the expression of $[Ca^{2+}]_{in}$-related proteins with different treatments. (**A** and **B**) Real-time recording of intracellular Ca^{2+} level without UCZNs on DRG neurons under NIR irradiation. DRG neurons were labeled by Fluo-4 AM (Scale bar, 50 μm). (**C**) Representative Western blots of CaM and CaMKII in PC12 cells with different treatments.

Groups	UCZNs (μg/mL)	Length of motor neuron axon (μm) (mean ± S.D.)	Protective effect
Control	-	142 ± 4.91	-
Model	-	94 ± 3.51	-
UCZNs + 980	40	114 ± 5.23 ***	42 % ***

table S2. The protective effect of UCZNs on SCI of zebrafishes, n = 6, ***P < 0.001.

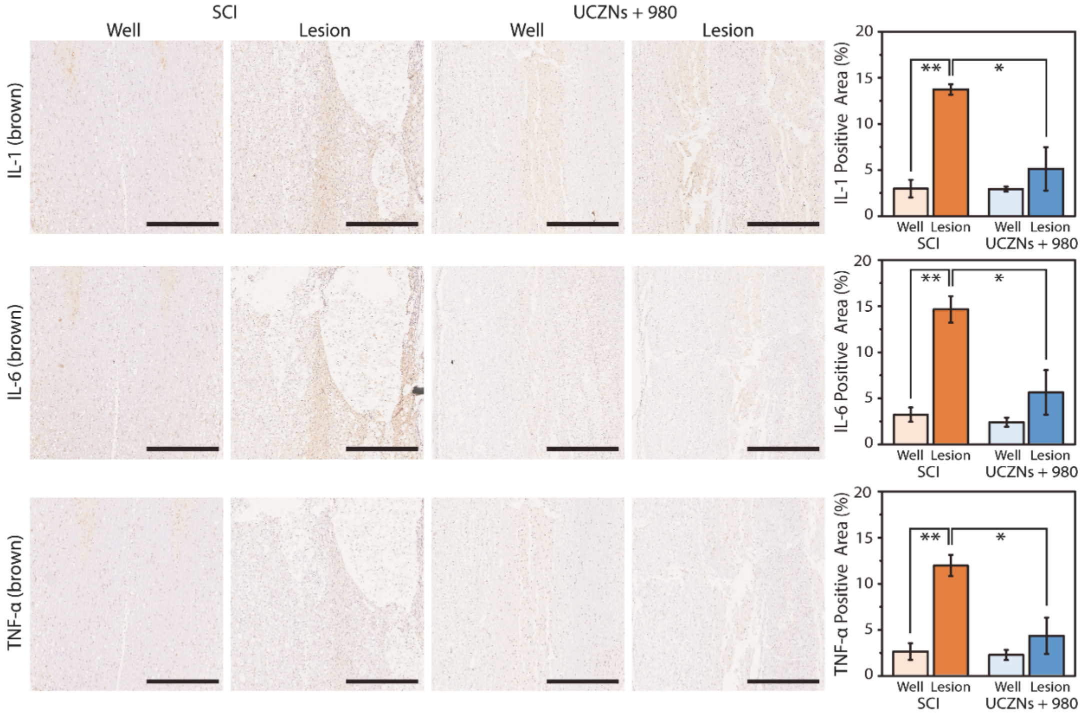

fig. S8. The inhibition effects of UCZNs with NIR irradiation on inflammatory. Representative immunohistochemical images and the positive area analysis of IL-1, IL-6 and TNF-α at both the lesion and the well region: without or with UCNPs + 980 treatment (scale bar, 500 μm) (n = 3, mean ± S.D.). Cell nuclei were stained with hematoxylin in blue. Significant was set at *P < 0.05 and **P < 0.01 analyzed by two-way ANOVA with Tukey's multiple comparisons test.

Legends for Movies:

movies S1. Colocation of PC12 cells and UCZNs. The co-location of UCZNs and PC12 cells. PC12 cells was labeled by Calcein-AM in green and UCZNs was excited by 980 nm laser emitting blue-violet light.

movies S2. NIR-triggered NO release from UCZNs. Real-time recording of NIR light triggered NO-releasing with or without UCZNs on PC12 cells *in vitro*. PC12 cells were labeled by DAF-FM DA.

movies S3. Growth of PC12 cells within 48 h. Real-time recording of the states of differentiation and neurite growth on PC12 cells with 40 μg mL^{-1} UCZNs, stimulated by NIR light within 48 h.

movies S4. Repair of DRG neurons. Visualization of the axon repair in DRG neurons with UCZNs activated by 980 nm laser.

movies S5. Calcium influx. Real-time recording of intracellular Ca^{2+} level with UCZNs on DRG neurons under NIR. DRG neurons were labeled by Fluo-4 AM.
